# Reprogramming of the *Hevea brasiliensis* Epigenome and Transcriptome in Response to Cold Stress

**DOI:** 10.3389/fpls.2022.831839

**Published:** 2022-03-21

**Authors:** Xiao Tang, Yonglei Zhang, Hong-Mei Yuan, Jinling Zhai, Xi Huang

**Affiliations:** ^1^Hainan Yazhou Bay Seed Laboratory, Sanya Nanfan Institute of Hainan University, Sanya, China; ^2^Hunan Rice Research Institute, Hunan Academy of Agricultural Sciences, Changsha, China

**Keywords:** *Hevea brasiliensis*, cold, DNA demethylation, transposon, RRBS

## Abstract

Low temperature is a key factor limiting the rubber plantation extending to high latitude area. Previous work has shown that cold-induced DNA demethylation was coordinated with the expression of cold-responsive (*COR*) genes in *Hevea brasiliensis*. In this work, reduced representation bisulphite sequencing analysis of *H. brasiliensis* showed that cold treatment induced global genomic DNA demethylation and altered the sequence contexts of methylated cytosines, but the levels of mCG methylation in transposable elements were slightly enhanced by cold treatment. Integrated analysis of the DNA methylome and transcriptome revealed 400 genes whose expression correlated with altered DNA methylation. DNA demethylation in the upstream region of gene seems to correlate with higher gene expression, whereas demethylation in the gene body has less association. Our results suggest that cold treatment globally change the genomic DNA methylation status of the rubber tree, which might coordinate reprogramming of the transcriptome.

## Introduction

Low temperature is a major environmental stress that seriously affects plant development and geographic distribution. Depending on the extent of the cold sensitivity of plants, cold stress can be subdivided into chilling (0–15°C) and freezing (<0°C; Wani et al., 2013). Chilling disrupts plant growth physiology by inducing photosynthesis-associated damage, chlorosis, apoptosis, and loss of membrane fluidity. Freezing causes ice formation and protoplast dehydration that directly kill cells. Plants exhibit increased freezing tolerance upon long-term exposure to non-freezing temperatures, a process known as cold acclimation (Liu et al., 2017a). Cold tolerance relies on many changes in plant processes, ranging from gene expression to physiological, biochemical, and metabolic processes (Knight and Knight, 2012; Theocharis et al., 2012). Cold acclimation activates rapid expression of the C-repeat binding factor (CBF) transcription factors and induces CBF-targeted genes, such as cold-responsive (*COR*) genes, which contribute to enhanced freezing tolerance (Jaglo-Ottosen et al., 1998; Fowler and Thomashow, 2002). Cold acclimation also increases the cytoplasmic volume and levels of organic acids (alpha-ketoglutarate, citrate, fumarate, and malate; Strand et al., 1999). During cold acclimation, the suppression of photosynthesis and photosynthesis gene expression is removed in leaves and is accompanied by increase in the activities of enzymes in the Calvin cycle and in the sucrose biosynthesis pathway (Strand et al., 1999).

Epigenetic regulation *via* mechanisms including DNA methylation and histone modification is involved in plant stress responses (Chinnusamy and Zhu, 2009a). Cytosine methylation is an important epigenetic mechanism that regulates gene expression and transposable elements (TEs) silencing and safeguards genome stability (Law and Jacobsen, 2010). In plant genomes, cytosine can be methylated in three sequence contexts: symmetric contexts CG and CHG (H = A, T, or C), or asymmetric context CHH (Law and Jacobsen, 2010; Vanyushin and Ashapkin, 2011). Both symmetric- and asymmetric-methylations are associated with repressive chromatin in gene promoters and with repression of gene transcription. Whole-genome DNA methylome analyses revealed that heavy cytosine methylation occurs in repetitive sequences and TEs (Penterman et al., 2007; Cokus et al., 2008). Methyltransferases Domains Rearranged Methylase 1 (DRM1) and DRM2 catalyze *de novo* cytosine methylation through the RNA-directed DNA methylation (RdDM) pathway, while the maintenance of symmetric CG and CHG methylation is mediated by the DNMT1-like enzyme MET1 and the plant-specific enzyme Chromomethylase 3 (CMT3), respectively (Cao and Jacobsen, 2002; Henderson and Jacobsen, 2007).

Plant cold response involves in epigenetic regulation. Vernalisation is a well-known example of epigenetic regulation in the plant cold response (Gendall et al., 2001; Bastow et al., 2004; Sung and Amasino, 2004). For other example, cold induced expression of *ZmMI1* was correlated with a reduction in methylation in the DNA of the nucleosome core in maize roots (Steward et al., 2002). In tobacco, cold stresses induced-DNA demethylation in the coding sequence of a glycerophosphodiesterase-like protein (*NtGPDL*) gene correlated with *NtGDPL* gene expression (Choi and Sano, 2007). However, cold induced epigenetic regulation is still relatively undocumented.

*Hevea brasiliensis* Muell. Arg. is an economically important tree originating from the tropical Amazon rain forest. When the locations of rubber plantations are extended to high latitude areas, such as southern China and northern Vietnam, cold stress becomes a key factor limiting rubber production. Low temperature prevents the tapping of trees for 1–3 months per year in the suboptimal areas (Rao et al., 1998; Jacob et al., 1999). Much attention has been paid to breeding cold-tolerant cultivars and studying the chilling physiology of *H. brasiliensis* (Rao et al., 1998; Mai et al., 2009, 2010), but the cold response mechanisms of *H. brasiliensis* have not been well elucidated. In a previous report, we showed that cold treatment altered the expression patterns of DNA methylation-associated genes, such as *HbMET*, *HbCMT*, and *HbDRM*. Long-term cold treatment induced DNA hypomethylation in the promoters of *HbICE1*, *HbCBF2*, and *HbMET*. Under natural conditions, the *HbICE1* and *HbMET* promoters switch from hypomethylation to hypermethylation status as the seasons change from winter to summer (Tang et al., 2018). *COR* gene expression levels were correlated with the status of DNA demethylation but not with the genetic background; we therefore proposed that epigenetic modification was closely related to the cold response of *H. brasiliensis* (Tang et al., 2018). However, the key question of how genome-wide DNA methylation in *H. brasiliensis* is affected by cold stress conditions remains open. Understanding this response might help us to better understand the mechanisms of cold acclimation in *H. brasiliensis*. In this study, we use reduced representation bisulphite sequencing (RRBS) to examine the alteration of the epigenome in response to cold stress. RRBS is a cost-effective method for rapid and affordable genome-wide DNA methylation analysis (Gu et al., 2011; Tanas et al., 2017). Additionally, we used digital gene expression (DGE) analysis based on RNA sequencing (RNA-seq) to analyze the expression of *COR* genes. Combined RRBS and DGE analyses allowed us to identify the causal link between genome-wide changes in DNA methylation and gene expression.

## Materials and Methods

### Plant Materials and Treatments

To minimize the variation of the genomic background, tissue-cultured self-rooting juvenile clones (JCs) from the same donor clones Reyan 7–33-97 were selected in the study (Li et al., 2016). Reyan 7–33-97 is an elite cultivar widely planted in China and its genome has been sequenced and fine assembled (Tang et al., 2016). The 60-day old JCs saplings (after transferring from sterile tube to polybag) were transferred to climate chamber for cold treatment as reported previously (Tang et al., 2018). Twenty saplings were treated under 19°C with the following growth conditions: 16-h light (100 lux)/8-h dark, 75% relative humidity for 1 month. Twenty control saplings were cultivated under 28°C with the same lighting, photoperiod, and relative humidity conditions. The cold-treated saplings were transferred to 28°C for a 1-month recovery and then further transferred to 19°C for the second and third treatments. A one-month interval of recovery between two treatments could prevent the saplings from leaf falling. After the third treatment, the treated and control leaf samples were collected, frozen immediately, ground in liquid nitrogen, and stored at −80°C until RNA and DNA extraction, respectively (Xia et al., 2011). Three independently treated leaf samples (treated or control) were mixed for pooling sample for RNA-seq and bisulphite sequencing.

### Reduced Representation Bisulphite Sequencing

After genomic DNAs were extracted from the pooling leaf samples, DNA concentration and integrity were detected by NanoPhotometer^®^ spectrophotometer (IMPLEN, CA, United States) and agarose gel electrophoresis, respectively, (Tang et al., 2018). The qualified DNA was digested by restriction endonucleases (*Msp*I) and repaired by 3′-end addition and adaptor ligation. The 40–220 bp fragments were selected for bisulfite conversion with Methylation-Gold kit (ZYMO, CA, United States). The converted DNA was PCR amplified and measured using an Agilent 2,100 bioanalyzer instrument, then the RRBS library was constructed and subjected the Illumina X10 sequencing platform with paired-end 150 bp sequencing by Gene Denovo Biotechnology Co. (Guangzhou, China). To get high quality clean reads, raw reads were filtered according to the following rules: (1) removing reads containing more than 10% of unknown nucleotides (*N*); (2) removing low quality reads containing more than 40% of low quality (*Q*-value ≤ 20) bases. The obtained clean reads were mapped to the Clone 7–33-97 reference genome using BSMAP software (Xi and Li, 2009). Then a custom Perl script was used to call methylated cytosines and the methylated cytosines were tested with the correction algorithm described (Lister et al., 2009). The methylation level was calculated based on methylated cytosine percentage in the whole genome, in each scaffold and in different regions for each sequence context (CG, CHG, and CHH). To assess different methylation patterns in different genomic regions, the methylation profile at flanking 2 kb regions and genebody (or TEs) was plotted based on the average methylation levels for each window.

### Differentially Methylated Regions Analysis

Differentially methylated regions for each sequence context (CG, CHG, and CHH) between two samples were identified according to the following criteria: (1) more than five methylated cytosines in at least one sample; (2) more than 10 reads coverage for each cytosine, and more than four reads for each methylated cytosine; (3) region length is between 40 bp and 10 kb; (4) the distance between adjacent methylated sites <200 bp; (5) the fold change of the average methylation level > 2; (6) Pearson’s chi-square test (*χ*^2^) value *p* ≤ 0.05. The putative DMRs overlapping at adjacent 2 kb (upstream or downstream) or body regions of genes or TEs were sorted out for further study (Akalin et al., 2012).

### Functional Enrichment Analysis of DMC/DMR-Related Genes

To analyze functional enrichment of genes affected by DMRs, Gene Ontology (GO) enrichment analysis and KEGG pathway enrichment analysis were conducted for DMR-related genes. GO enrichment analysis provides all GO terms that significantly enriched in genes comparing to the genome background, and filter the genes that correspond to biological functions. Firstly, all genes were mapped to GO terms in the Gene Ontology database.^1^ KEGG is the major public pathway-related database.^2^

### RNA Sequencing

After total RNA was extracted, mRNA was enriched by Oligo (dT) beads. Then the enriched mRNA was fragmented into short fragments and reverse transcripted into cDNA with random primers. Second-strand cDNA were synthesized, purified with QiaQuick PCR extraction kit, and then ligated to Illumina sequencing adapters. The ligation products were size selected by agarose gel electrophoresis, PCR amplified, and sequenced using Illumina HiSeqTM 2,500 by Gene Denovo Biotechnology Co. (Guangzhou, China). High quality clean reads were further filtered according to the following rules: (1) removing reads containing adapters; (2) removing reads containing more than 10% of unknown nucleotides (*N*); and (3) removing low quality reads containing more than 50% of low quality (*Q*-value ≤ 20) bases.

The clean reads were then mapped to reference genome of Clone 7–33-97 by TopHat2 (version 2.0.3.12; Kim et al., 2013; Tang et al., 2016). The alignment parameters were as follows: (1) maximum read mismatch is 2; (2) the distance between mate-pair reads is 50 bp; and (3) the error of distance between mate-pair reads is ±80 bp. After aligned with reference genome, unmapped reads (or mapped very poorly) were then re-aligned with Bowtie2, the enriched unmapped reads were split into smaller segments which were then used to find potential splice sites. Splice sites were built with initial unmapped reads by TopHat2 without relying on the known genes annotation (Trapnell et al., 2010). The reconstruction of transcripts was carried out with software Cufflinks (Trapnell et al., 2012), which together with TopHat2, allow to identify new genes and new splice variants of known reference genes. EdgeR software was used to normalize the data and extract digital gene expression (DGE) data (Robinson et al., 2010). Differentially expressed genes (DEGs) were selected when the false discovery rate (FDR) was <0.05. DGE data were used for clustering of DMRs. Gene Ontology (GO) term and Kyoto Encyclopedia of Genes and Genomes (KEGG) pathways analyses were visualized with Pathview software (Young et al., 2010; Wang et al., 2015).

### Integrated Analysis of DNA Methylation and the Transcriptome

After differentially Methylated Regions (DMRs) and DEG analysis, the distribution of Spearman rho statistics between DNA methylation and gene expression was calculated including either all pairs of genes/methylation positions or only pairs formed by a DMP and a DEG. All DNA methylation and transcriptomics data analyzed were output in Bam format.^3^ A list of genes and transposons and the corresponding DNA methylation positions was obtained, and a correlation coefficient was calculated for each pair. The average DNA methylation levels of the genes and TEs in the control and cold-treated plants were displayed in a Bam format file. All data were visualized and analyzed using box plots, heat maps, and circos plots.

### Validation of Differential Gene Expression by Quantitative RT-PCR

Quantitative RT-PCR (qRT-PCR) was used to confirm differential expression of the unigenes obtained from sequencing and to further analyze the reliability of the RNA-seq data generated in this study. Primers used in this work are listed in Supplementary Table S6. The *HbACT7b* gene was used as an internal control. First-strand cDNA was synthesized using a cDNA synthesis kit according to the manufacturer’s instructions (Fermentas, Vilnius, Lithuania). qRT-PCR was performed according to Li’s method (Li et al., 2014). All relative expression data were analyzed using GraphPad Prism 7 software. Each biological sample was represented by three independent replicates. qRT-PCR conditions were as follows: denaturation at 95°C for 30 s followed by 50 cycles for 10 s at 94°C, 30 s at 60°C, and 15 s at 72°C for amplification. Data obtained from qRT-PCR analysis were clustered in accordance with the instructions provided by Stratagene (Santa Clara, CA, United States). The expression analysis was performed from three individual reactions.

## Results

### Cold Treatment Induced Global DNA Demethylation in *H. brasiliensis*

After cold treatment, genomic DNA was isolated from leaf samples for RRBS. The sequencing statistic was shown in Table 1 and Supplementary Table S1. There were 83,531,456 and 82,345,180 clean reads generated by RRBS for control and cold treated samples, respectively (Supplementary Table S1). After filtering, 7,900 MB of clean data and 82,143,730 clean reads were generated from the control samples, 91.24% of which were uniquely mapped to genomic sequences of *H. brasiliensis* cultivar Reyan7-33-97 (NCBI SRA data: PRJNA741882). In total, 7,766 MB of clean data and 81,565,518 clean reads were generated from the cold-treated samples, 94.80% of which were uniquely mapped (Table 1 and Supplementary Table S1).

**Table 1:** Reduced representation bisulphite sequencing (RRBS) statistics.

Sample	Clean reads/clean data (bp)	GC content (%)	Mapping rate (%)	mCG no./proportion	mCHG no./proportion	mCHH no./proportion	Total methylated
Control	82,143,730/7,900,525,750	24.58%	91.24%	1,610,060/23.78%	2,579,031/38.08%	2,582,602/38.14%	6,671,693
Cold-treated	81,565,518/7,766,520,664	24.56%	94.80%	632,899/26.69%	939,769/39.64%	798,268/33.67%	2,370,936

Sequencing depth analysis showed that the coverage level in genomic functional elements ranged from 21 to 51%, in which the coverage rate of C, CG, CHG, and CHH were indicated, respectively (Supplementary Table S3). RRBS results also showed that the numbers of the mCG、mCHG and mCHH in the control samples were 1,610,060, 2,579,031, and 2,582,602, respectively (23.78, 38.08, and 38.14%, respectively). By contrast, the methylation numbers of the cold-treated samples were 632,899, 939,769, and 798,268, respectively (26.69, 39.64, and 33.67%, respectively). These data indicated that cold treatment strongly decreased the methylation number of mCG, mCHG, and mCHH. The total number of three types methylated cytosines decreased from 6,771,693 (control) to 2,370,936 (cold), suggesting that average DNA methylation level were down regulated by cold treatment (Table 1 and Supplementary Table S4).

**Figure 1:**
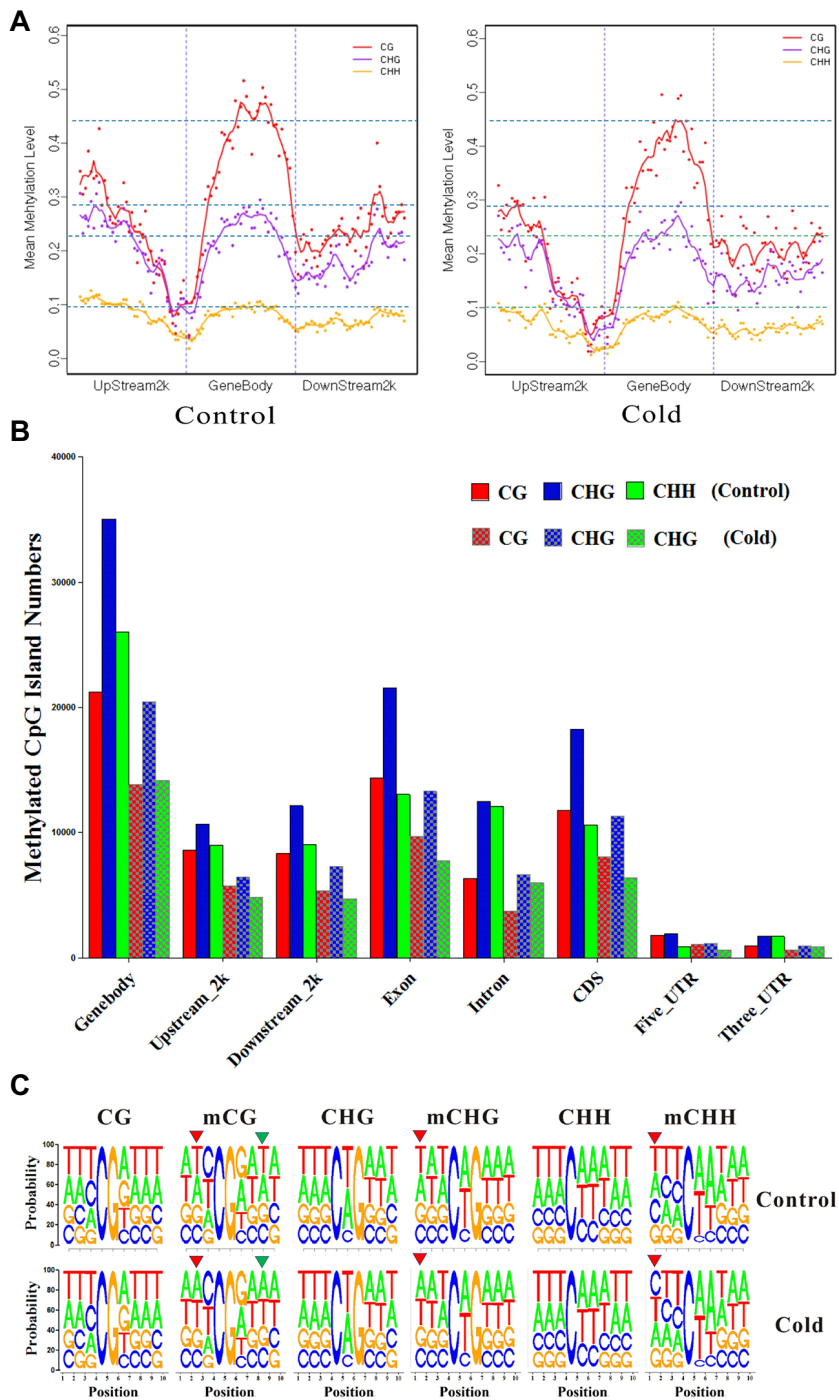
Distribution of methylated cytosines in genic regions and sequence preference after cold treatment. **(A)** Methylation levels of CG, CHG, and CHH distributed in the gene body, upstream region, and downstream region. Upstream, 2-kb region upstream of the transcription start site; gene body; downstream, the 2-kb region downstream of the transcription termination site. **(B)** Distribution of mCG, mCHG and mCHH in the gene body, 2-kb upstream region, 2-kb downstream region, exon, intron, CDS, 5-UTR, and 3-UTR. Solid column and squares column histograms represent the control and cold-treated samples, respectively. The data were normalized by the mapping rate and mapping reads. **(C)** Logos of sequence contexts that are preferentially unmethylated/methylated CG, CHG, and CHH in control and cold-treated samples in which the cytosine is in the fifth position. The logo graphically displays the sequence enrichment at a particular position in the alignment of 10-mers in each class.

Further analysis of the distribution of methylated cytosines in various gene regions revealed that the level of mCG methylation was much higher than those of the other two types of Methylated cytosines. Lower methylation was observed near the transcription start site. The levels of mCG methylation located in the gene body, 2-kb upstream region, and 2-kb downstream region were evidently decreased in the cold-treated samples. mCHG methylation was lower than mCG methylation but higher than mCHH methylation. Cold treatment slightly induced demethylation of mCHG and mCHH in the 2-kb upstream and downstream regions (Figure 1A). When gene regions were further subdivided into the gene body, 2-kb upstream region, 2-kb downstream region, exon, intron, coding sequence (CDS), 5′-untranslated region (UTR), and 3′-UTR, all methylation levels distributed among the mCG, mCHG, and mCHH were lower in the cold-treated samples than in the control samples (Figure 1B), suggesting that cold treatment induced global DNA demethylation in *H. brasiliensis*.

Statistics showed that cold treatment induced significant alteration of the sequence context upstream of methylated cytosines (e.g., mCG, mCHG, and mCHH) and the sequence context downstream of mCG, whereas no difference was found in the sequence context of unmethylated cytosines after cold treatment (Figure 1C). These results suggest that cold treatment reprogrammed the *H. brasiliensis* epigenome.

### Cold Treatment Enhanced mCG Methylation in TEs

**Figure 2:**
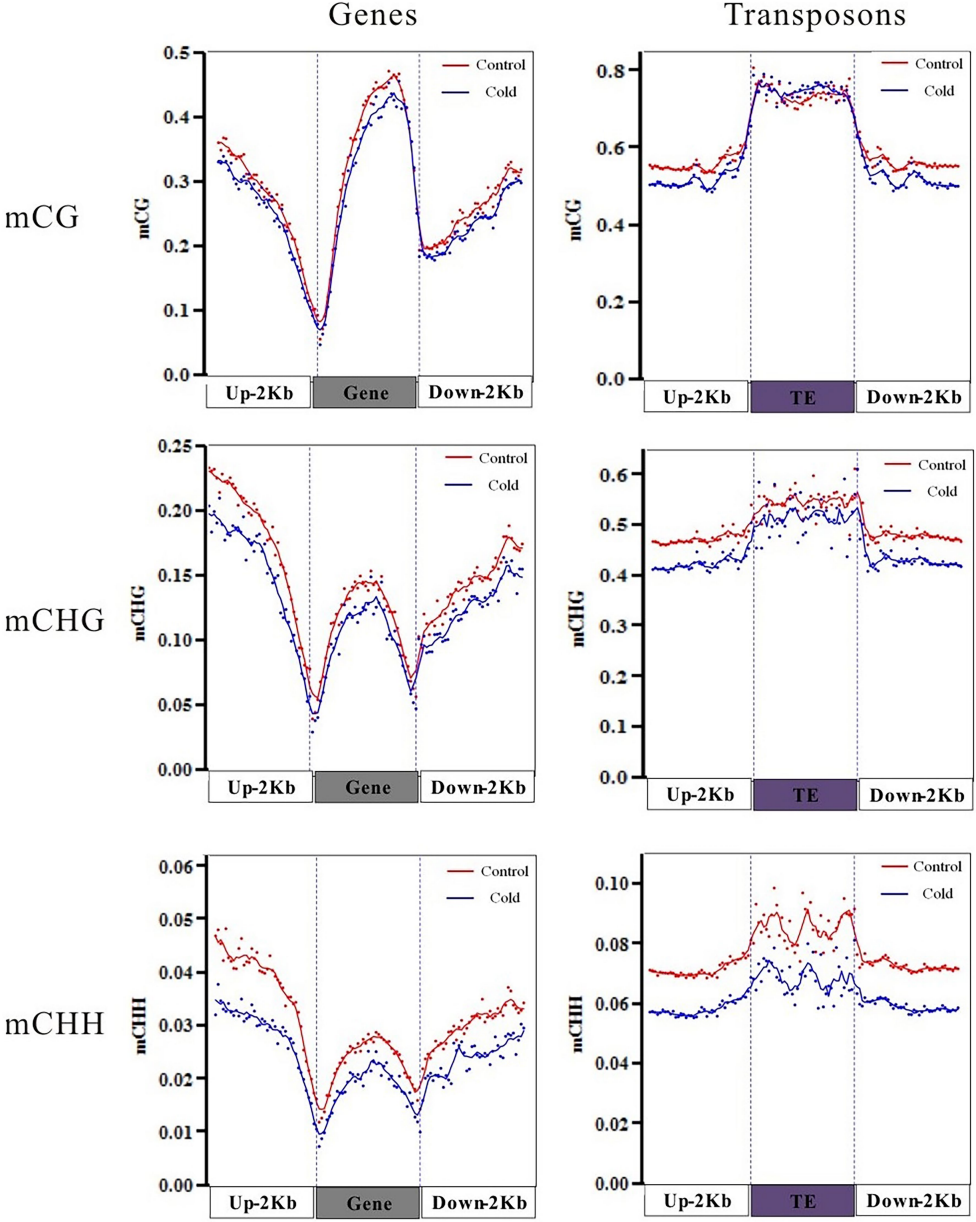
Methylation patterns of genic and transposon element (TE) regions after cold treatment. Levels of mC, mCG, mCHG, and mCHH methylation distributed in the gene body, upstream region, and downstream region of genes and TEs. 2-kb region upstream of the transcription start site; gene body; downstream, the 2-kb region downstream of the transcription termination site.

To reveal the differential methylation patterns between functional genes and TEs, which were annotated in *Hevea brasilensis* genomic sequences (Tang et al., 2016), statuses of mCG, or mCHG, or mCHH between control and cold treatment were compared, respectively. The distribution of methylation in genes exhibited a “W” shape. Higher methylation levels showed in the gene body, 2-kb upstream region, and 2-kb downstream region but markedly lower level at the transcription start site and transcription termination site (Figure 2, left panel). By contrast, the distribution of methylation in TEs exhibited a “single-hump camel” profile. The DNA methylation level changed dramatically at the TE boundaries, whereas both the upstream and downstream regions had lower methylation levels (Figure 2, right panel). Similar phenomenon was also observed in *Brassica rapa* (Chen et al., 2015).

DNA methylation plays an important role in modulating TE silencing. The DNA methylation level is much higher in TEs than in genic regions. Cold treatment reduced average DNA methylation levels in mCG, mCHG, and mCHH in genic regions, including the gene body, 2-kb upstream region, and 2-kb downstream region (Figure 2, left panel). Interestingly, cold treatment slightly increased the mCG methylation levels in gene body of TEs, although mCHG and mCHH methylation levels were significantly decreased by cold treatment (Figure 2, right panel). These differential methylation patterns might reflect the distinct roles of genes and TEs in the *H. brasiliensis* mechanisms for coping with cold stress.

### Digital Gene Expression Analysis of Cold-Treated *H. brasiliensis*

To gain a global view of the gene expression patterns induced by the cold treatment of *H. brasiliensis*, we used RNA-seq to identify differentially expressed genes. In total, 3,764,205,386 and 3,433,710,574 HQ (high quality) clean data were acquired from the control and cold-treated samples, respectively (Supplementary Table S2, NCBI SRA data: PRJNA741882). Genes coverage analysis showed that the RNA-seq for control sample covers 70.55% of Reyan7-33-97 reference genes at 80–100% coverage rate and 14.85% genes at 60–80% coverage rate (Supplementary Figure S1A); RNA-seq for cold treated sample has similar coverage rate (Supplementary Figure S1B), suggesting that RNA-seq data have relative higher coverage. Statistics of RNA-Seq reads showed that 29131and 30,242 genes were mapped to the genes of Reyan7-33-97 for control and cold treated samples, respectively (Supplementary Table S5), of which 5,736 genes showed differential expression (low FDR < 0.001 and *p*-value <0.05), including 3,693 upregulated genes and 2043 downregulated genes (Supplementary Figure S1C). Scatter plots indicated that most data points were distributed near the diagonal, suggesting that the expression levels of the majority of genes were largely unaffected by cold treatment (Supplementary Figure S1D).

**Figure 3:**
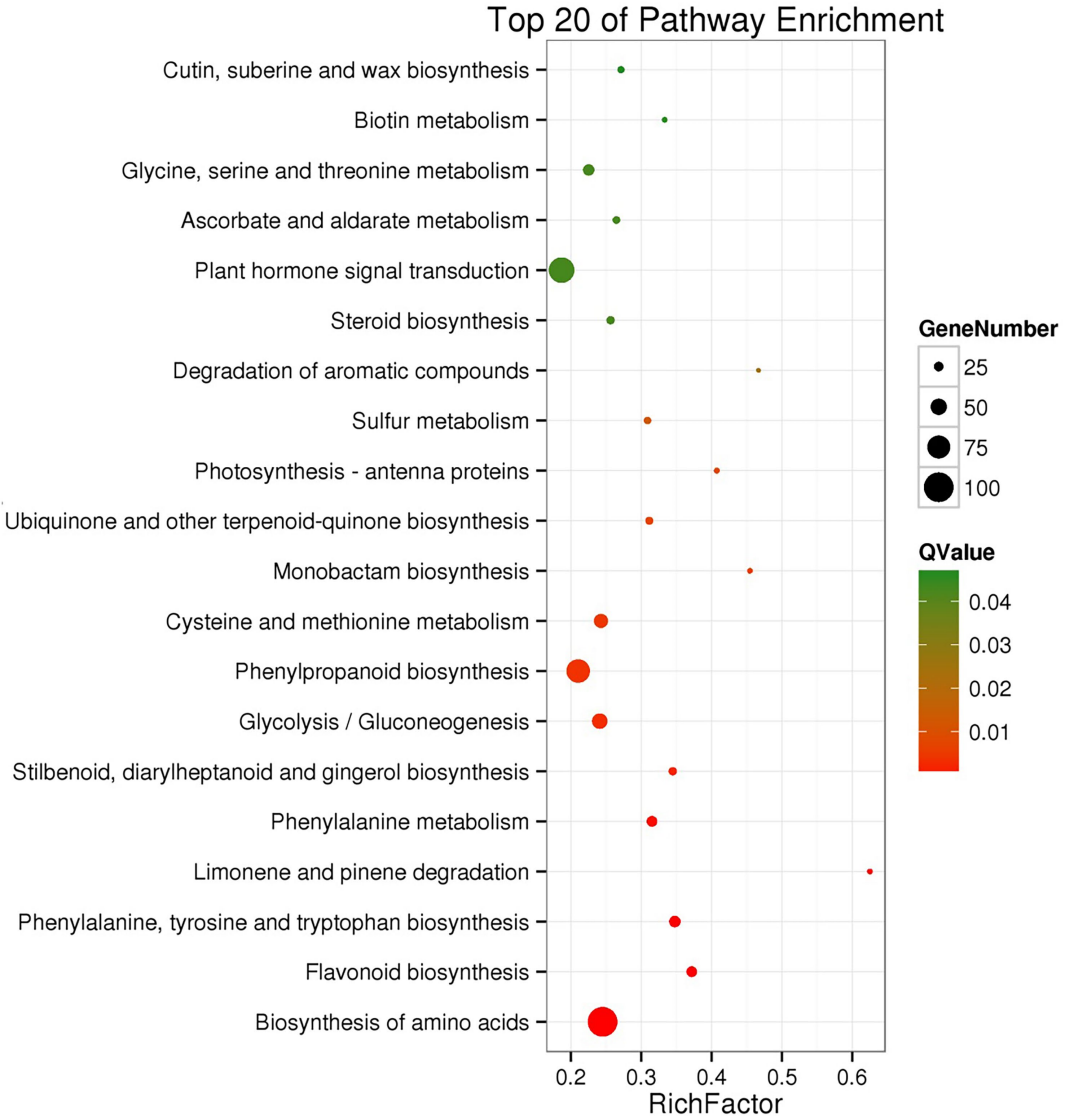
KEGG pathway enrichment of differentially expressed genes after cold treatment. The solid circles represent differentially expressed genes located in the pathway. The larger of the solid circle represents the RichFactor enrichment level of the class. The *Q*-value is a value of *p* after a multiple hypothesis test correction from 0 to 1.

To identify pathways in which the cold-induced differentially expressed genes (DEGs) might be involved, the DEGs were categorized based on KEGG pathways and GO term enrichment. The cold-induced DEGs were enriched in the KEGG pathways of plant hormone signal transduction, phenylpropanoid biosynthesis, and amino acid biosynthesis (Figure 3). The DEGs were enriched in 47 GO terms, among which “catalytic activity,” “metabolic process,” “cellular process,” “single-organism process,” and “binding” were the most enriched (Supplementary Figure S2).

### Integrated Analysis of DEGs and Changes in DNA Methylation

**Figure 4:**
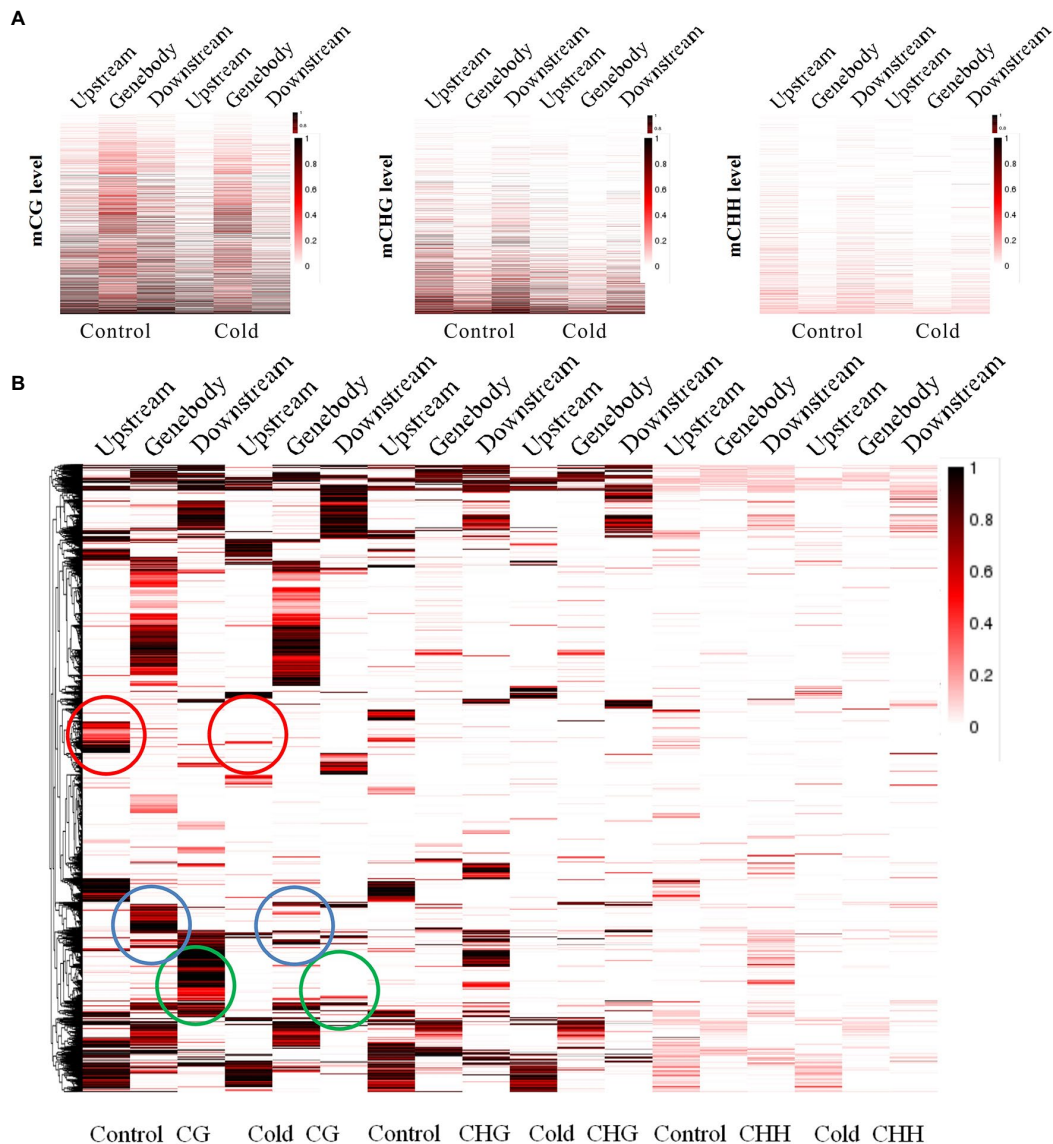
Distribution and clustering of the methylation of differentially expressed genes. **(A)** The methylation levels of mCG, mCHG and mCHH in control and cold-treated samples are shown by a heat map (upper panel). The box plot shows CG, CHG, and CHH methylation levels at hyper-DMRs. Dark horizontal line, median; edges of boxes, 25th (bottom) and 75th (top) percentiles; whiskers, minimum and maximum percentages of DNA methylation (lower panel). **(B)** Heat map of gene clustering representing the methylation levels of differentially expressed genes at various gene regions. The methylation level is indicated by the depth of the color.

**Figure 5:**
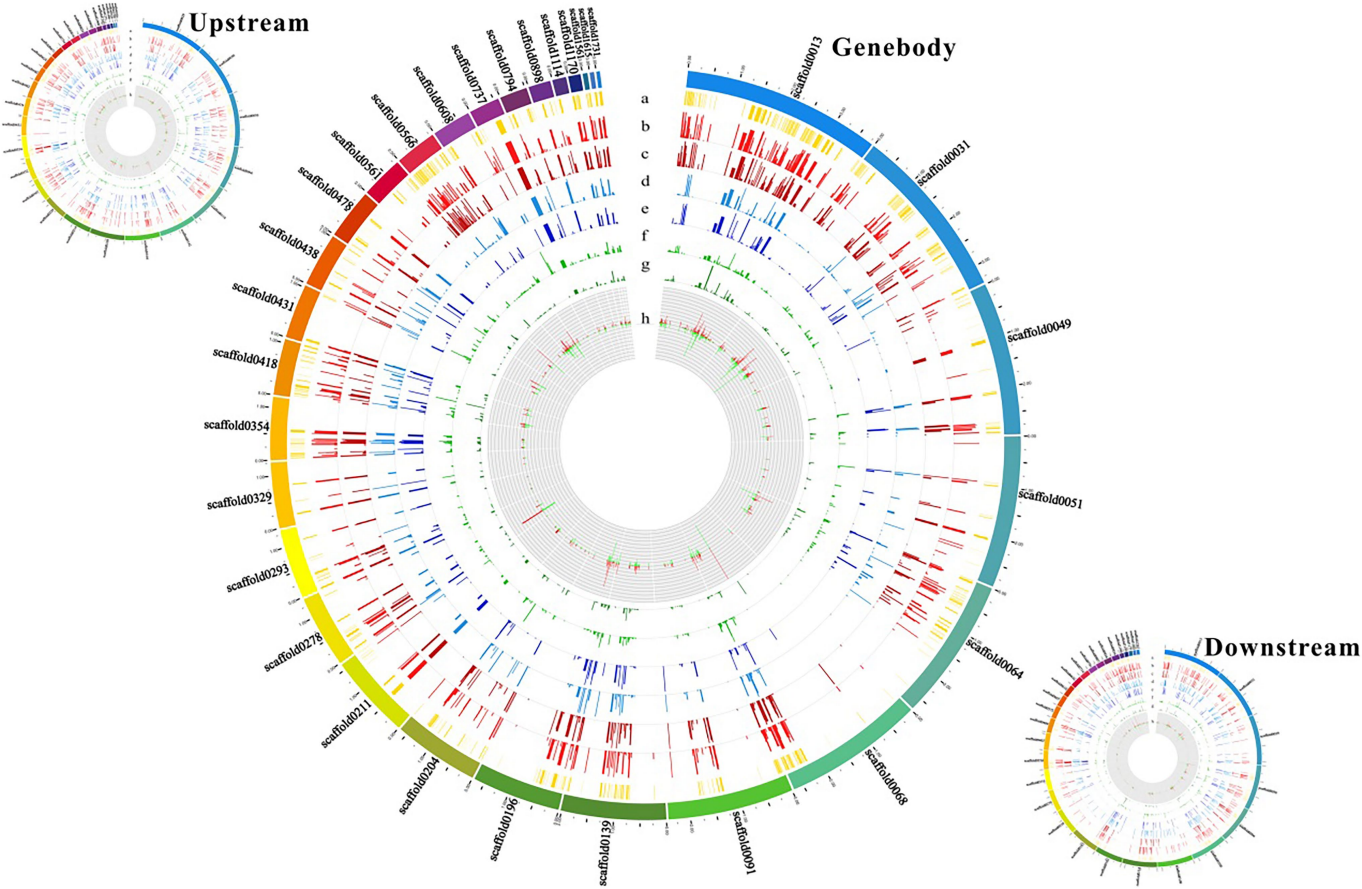
Circos plot showing methylation sites and gene expression levels at the genomic level. Outer rings represent different scaffolds. (a) Yellow column represents the base site where the methylation takes place. (b) Red column represents the mCG methylation site in the control sample. (c) Dark red column represents the mCG methylation site in the cold-treated sample. (d) Blue column represents the mCHG methylation site in the control sample. (e) Dark blue column represents the mCHG methylation site in the cold-treated sample. (f) Green column represents the mCHH methylation site in the control sample. (g) Dark green column represents the mCHH methylation site in the cold-treated sample. (h) Up- and down–regulation of gene expression levels after cold treatment indicated by red and green colors, respectively.

To establish a correlation between RRBS and DEGs induced by cold treatment, we engineered the mutual information-based analysis using count data from the DGE analysis and overlaid the DNA methylation data by summarizing the methylation changes of the linked DMRs and DMPs. Additionally, to better understand the distribution of methylation levels among the cold-induced DEGs in DMRs, the methylation levels of the DEGs were visualized using a heat map. First, the methylation levels of mCG, mCHG, and mCHH of DEGs in control and cold-treated samples were shown by a heat map. Highest methylation level appeared in mCG, in which demethylation induced by cold could be observed in upstream, genebody and downstream regions. Although methylation level of mCHG and mCHH was much lower than mCG, cold treatment also evidently induced demethylation (Figure 4A). Cluster analysis using heat map to represent the methylation levels of DEGs reveals different patterns of methylation modification after cold treatment. The more genes showed demethylation under cold stress, some of which at upstream regions (marked with red rings) and some of which at genebody (marked with blue rings). Interestingly, some cluster of genes exhibited increased methylation level, e.g., at downstream regions (marked with green rings; Figure 4B). These results suggest that cold treatment induced different modifications of DNA methylation in different genes regions of rubber tree. To better visualize the combined DEGs and RRBS analysis, a circos plot of epigenomic changes superimposed with DEGs was generated. The methylation sites of mCG, mCHG, and mCHH, and the expression levels of the corresponding DEGs were visualized at the genomic level. The DEGs that correlated with alterations in DNA methylation were distributed among 30 different scaffolds, which enable us to globally observe the methylation status and gene expression at the genomic width (Figure 5).

**Figure 6:**
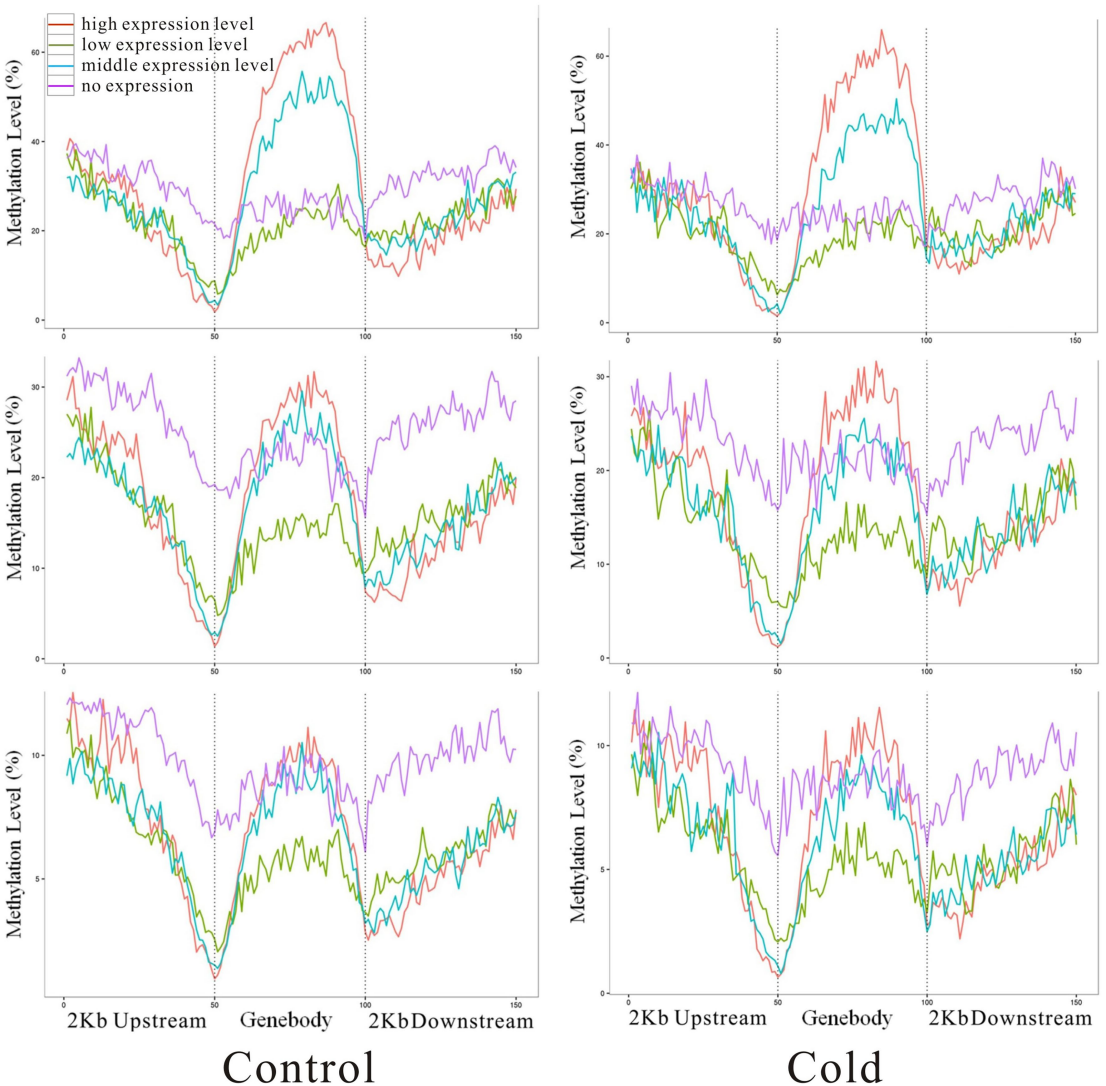
Correlation of methylated cytosines with gene expression levels. The methylation level is indicated on the *y*-axis, and *x*-axis indicates the regions of 2 kb up and downstream of genebody. Red, green, blue, and pink colors represent high, moderate, low, and no expression of genes, respectively.

Integrated analysis of DEGs and modifications to DNA methylation indicated that highly expressed genes (red line) showed higher methylation levels in the gene body but low methylation levels in the upstream and downstream regions (Figure 6). By contrast, genes that were not expressed (pink line) showed lower methylation levels in the gene body but higher methylation levels in the downstream region at mCG, mCHG, and mCHH. Methylation status in upstream region might correlate with gene expression, there are fewer reports about the role of downstream region in gene transcription regulation, although plenty documents have shown that 3′UTR involve in mRNA-based processes (Mayr, 2017, 2019). It is reasonable to speculate that 2 kb downstream region of one gene might include part or even complete upstream sequence of the next adjacent gene. DMR-related differentially expression genes was analyzed by GO annotation. These genes involve in many processes, such as protein modification, response to stimulus, metabolic process, macromolecular modification, and so on (Supplementary Table S7).

### Validation of Cold-Induced Genes by qRT-PCR

Circle mapping and whole-genome visualisation using the Integrative Genomics Viewer (IGV) software revealed that more than 400 genes exhibited expression levels that were correlated with methylation modification (Figure 6). We selected 23 of these genes for validation by qRT-PCR. These genes included a cold-regulated plasma membrane protein gene (*HbCRP1*), chloroplast genes (*HbCHL1/2*), dehydratase genes (*HbDEH1/2/3*), a histone demethylase gene (*HbHDM1*), zinc finger protein genes (*HbCZF2/VZF3*), a methyltransferase gene (*HbTMET1*), a sugar transporter gene (*HbSTE3*), glycosyltransferase genes (*HbUGT1/GT1*), an early-responsive to dehydration gene (*HbERD1*), NAC domain-containing protein genes (*HbNAC1/NAC2*), a dehydration-responsive element-binding protein gene (*HbDRE1*), transposons (*HbRTN1/2*), and ethylene-responsive transcription factor genes (*ERF*). The qRT-PCR analysis confirmed the cold treatment-induced expression of these genes shown by the DGE analysis (Supplementary Figure S3).

## Discussion

Being sessile organisms, higher plants inevitably endure more environmental stresses (e.g., cold, heat, UV irradiation, salinity, drought, and heavy metal) than animals in their life cycles (Puli and Raghavendra, 2012; Liu et al., 2017b). Of these abiotic stresses, cold stress is an important factor that limits plant range expansion and survival (Sanghera et al., 2011). When rubber tree was introduced to suboptimal planting areas, low temperature becomes the most detrimental factor influencing latex production and limits the expanding of rubber plantations to higher-latitude areas. All popular rubber tree cultivars are derived from “Wickham base,” and little genetic variation was observed among the different cultivars of *H. brasiliensis* (Tang et al., 2016). Despite their similar genetic makeup, rubber tree cultivars exhibit various levels of cold tolerance (Mai et al., 2009, 2010). Epigenetic modification induced by location-specific environmental factors has been reported in rubber trees (Uthup et al., 2011). Our previous reports showed that cold treatment switched *HbICE1* promoters from hypermethylation status to hypomethylation status under both artificial and natural conditions, cold-induced DNA demethylation of *COR* gene might play a key role in cold acclimation in rubber tree (Tang et al., 2018).

In contrast to whole-genome bisulphite sequencing, which requires deep re-sequencing of the entire genome (Cokus et al., 2008; Zhong et al., 2013), RRBS enriches CG-rich parts through digestion with *Msp*I (recognized context sequence C′CGG) and ligation of genomic DNA to adapters for bisulphite conversion and Illumina sequencing (Gu et al., 2011). This work demonstrated that RRBS is a cost-effective method for genome-wide DNA methylation analysis. RRBS revealed that cold treatment induced global DNA demethylation and significant genome-wide alteration of the sequence contexts of methylated cytosines in *H. brasiliensis*. Preexisting DNA methylation can be lost as a consequence of passive or active demethylation processes. The active removal of cytosine methylation is catalyzed by the members of the DNA glycosylase family (Chinnusamy and Zhu, 2009a,b). Previous report showed that the demethylation-associated genes were strongly induced by cold treatment (Tang et al., 2018), suggesting that cold induced global DNA demethylation might be an active process in rubber tree. Similar phenomenon was observed in other plant species. When maize seedlings were exposed to cold stress, a genome-wide demethylation occurred in root tissues (Steward et al., 2002). Rapid alterations in cytosine methylation occurred throughout the periods of chilling and freezing associated with the cold tolerance of an alpine subnival plant, *Chorispora bungeana* (Song et al., 2015). It is the first time to report that cold treatment globally reprogrammed the epigenome of *H. brasiliensis*. Following the global DNA demethylation, extensive chromatin rearrangement and gene expression regulation might occur. Integrated analysis of the DNA methylome and the transcriptome revealed the correlation of gene expression with altered DNA methylation. DMR-related differentially expression genes involve in protein modification, response to stimulus, metabolic process, macromolecular modification, and so on. These genes might regulate physiological, biochemical, and metabolic processes to accumulate protective proteins and sugars and modify lipid metabolism to cope with cold stress. However, how such a demethylation locates its target regions remains to be explored.

Another interesting finding is the differential pattern of cold induced DNA methylation in TEs. Total mCG methylation was enhanced by cold treatment in the TEs. The differential methylation patterns reflect the different roles that genes and TEs play in *H. brasiliensis* in coping with cold stress. TEs have long been known as useless “junk” DNA. These mysterious mobile elements of the genome might play some kind of regulatory role, determining which genes are turned on and when this activation takes place. TEs not only play a role in regulating gene expression, but also in generating different cell types and different biological structures (Pray, 2008). Enhanced methylation of TEs under cold stress influences the TEs transcription and decrease the “jumping” activity of TEs, which might affect the gene expression related to cold response. This phenomenon has not been described in previous reports. In contrast, hypomethylation and transposition of the Tam-3 transposon were induced by cold stress in *Antirrhinum majus* (Hashida et al., 2006). Repetitive elements were reported to be transcriptionally activated in a genotype-dependent manner in alfalfa during cold acclimation (Ivashuta et al., 2002).

Most of stress-induced epigenetic modifications are reset to the basal level once the stress is relieved, whereas some of the modifications may be stable. These epigenetic stress memories may help plants cope more effectively with subsequent stresses (Chinnusamy and Zhu, 2009a). Vernalisation is a well-documented “memory response” in which mCG methylation serves as a central epigenetic coordinator that ensures stable transgenerational inheritance (Mathieu et al., 2007). Integrated analysis of DEGs and changes in DNA methylation revealed several protective genes for tolerance to cold stress. *HbSTE3* is related to sugers transport and metabolism (Tang et al., 2010). *ERF* might involve in latex drainage related with ethylene signaling (Duan et al., 2013). *HbCRP1*, *HbERD1* and *HbDRE1* are well-known cold stress responsive genes of plant. NAC domain-containing protein gene (*HbNAC1/NAC2*) is involved in response to many abiotical stresses. These genes might help to maintain rubber production under cold stress.

A key question regarding the epigenetic regulation of the cold response in *H. brasiliensis* is whether cold-induced DNA demethylation is a transient or “memory” response. Under natural condition, the methylation status of *HbICE1* and *HbMET1* promoters changed seasonally (Tang et al., 2018), suggesting that these stress-induced epigenetic modifications were transient response. This work reveals a global cold induced DNA demethylation, it is still unknown whether it was transient or a stable “memory.” Clarification of this question might help us to answer whether cold acclimation by methylation modification could be applied to tree breeding and production. Additionally, the stress-induced change of DNA methylation is usually coordinated with histone modification, e.g., cold response in vernalisation, which requires the epigenetic silencing of FLOWERING LOCUS C (FLC) by histone modification (Bastow et al., 2004; Sung and Amasino, 2004). Whether cold acclimation in the rubber tree also involves in histone modification requires further investigation.

## Conclusion

Cold treatment induced global genomic DNA demethylation of *H. brasiliensis* and altered the sequence contexts of methylated cytosines, but increased the levels of mC methylation in TEs. Integrated analysis of the DNA methylome and transcriptome revealed 400 genes whose expression correlated with altered DNA methylation. DNA demethylation in the upstream region of gene seems to correlate with higher gene expression, whereas demethylation in the gene body has less association. Global change of the genomic DNA methylation status induced by cold treatment might coordinate reprogramming of the transcriptome in the rubber tree.

## Data Availability Statement

The datasets presented in this study can be found in online repositories. The names of the repository/repositories and accession number(s) can be found at: https://www.ncbi.nlm.nih.gov/genbank/, NCBI SRA data: PRJNA741882.

## Author Contributions

XT performed most of the experiments. YZ and H-MY performed the bioinformatics analysis. XH and JZ supervised and wrote the manuscript. All authors have read and approved the final version of the manuscript.

## Funding

This work was supported by the National Natural Science Foundation of China (grant numbers 31860194 and 31860222).

## Conflict of Interest

The authors declare that the research was conducted in the absence of any commercial or financial relationships that could be construed as a potential conflict of interest.

## Publisher’s Note

All claims expressed in this article are solely those of the authors and do not necessarily represent those of their affiliated organizations, or those of the publisher, the editors and the reviewers. Any product that may be evaluated in this article, or claim that may be made by its manufacturer, is not guaranteed or endorsed by the publisher.

## Footnotes

^1^ http://www.geneontology.org/

^2^ http://www.kegg.jp/kegg/

^3^ http://samtools.sourceforge.net/SAM1.pdf

## Abbreviations

CBF: C-repeat binding factor
COR: Cold-responsive
DEGs: Differentially expressed genes
DGE: Digital gene expression
DMCs: Differentially methylated cytosines
DMP: Differentially methylated position
DMR: Differentially methylated region
GO: Gene Ontology
KEGG: Kyoto Encyclopedia of Genes and Genomes
RRBS: Reduced representation bisulphite sequencing
TEs: Transposable elements
UTR: Untranslated region
