# Supplementary material for: Reprogramming of the Hevea brasiliensis Epigenome and Transcriptome in Response to Cold Stress
Source: Front Plant Sci. 2022 Mar 21;13:831839. doi: 10.3389/fpls.2022.831839 (PMC8979024; doi:10.3389/fpls.2022.831839)
Supplement: Supplementary file 1 [file Table_1.DOCX]

**Supplemental materials**


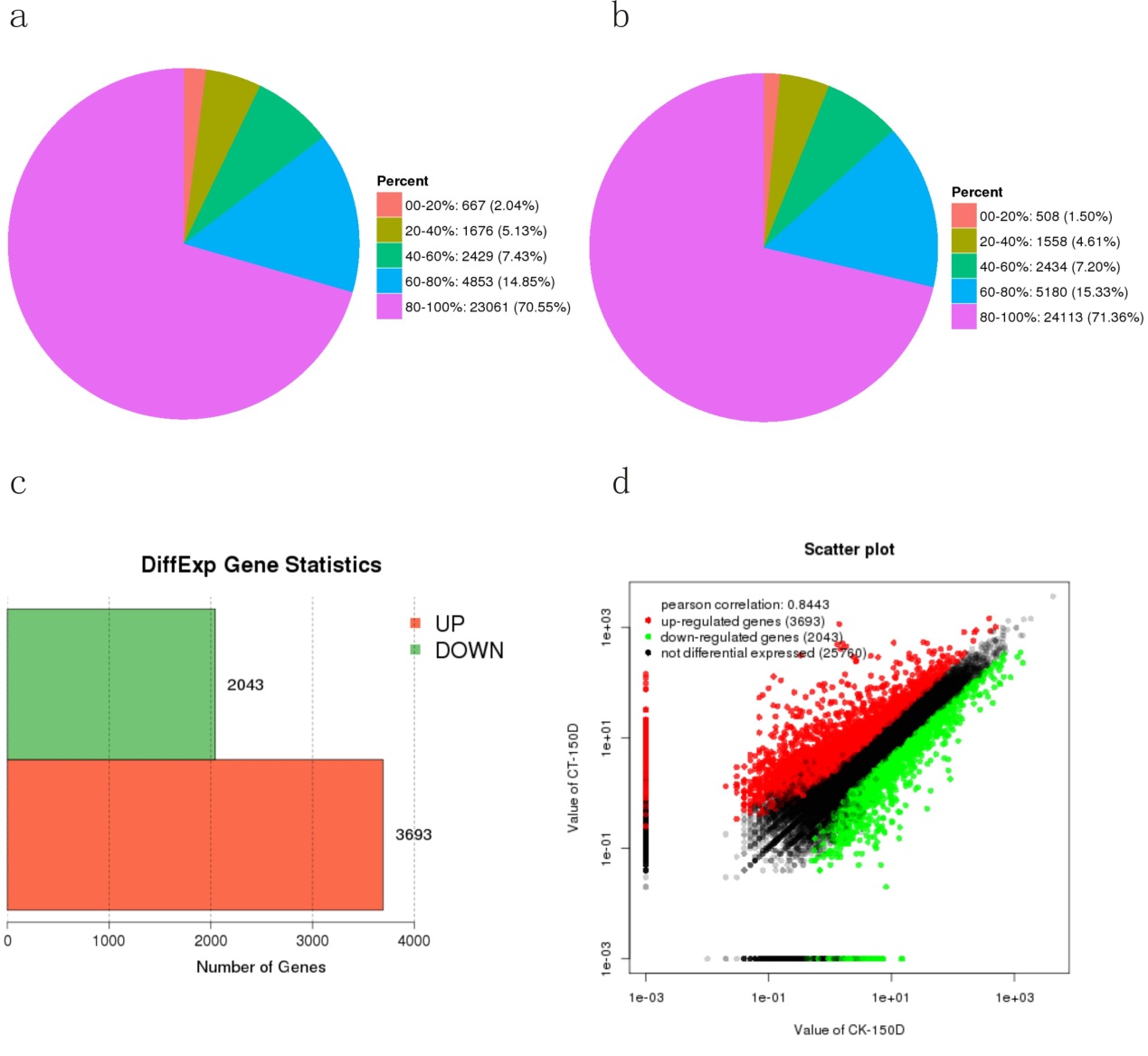


**Figure S1. Properties of differentially expressed genes identified by digital gene expression (DGE) analysis.** **a.** Distribution of genes coverage of control sample to Reyan7-33-97 reference genes. **b.** Distribution of genes coverage of cold treated sample to Reyan 7-33-97 reference genes. **c.** Numbers of differentially expressed genes after cold treatment. **d.** Scatter plot of the DGE profile generated from RNA-seq data. Vertical and horizontal axes represent levels of gene expression in control and cold-treated plants, respectively. Red dots and green dots indicate more than two-fold up- and down-regulated (FDR <0.05) gene expression, respectively.


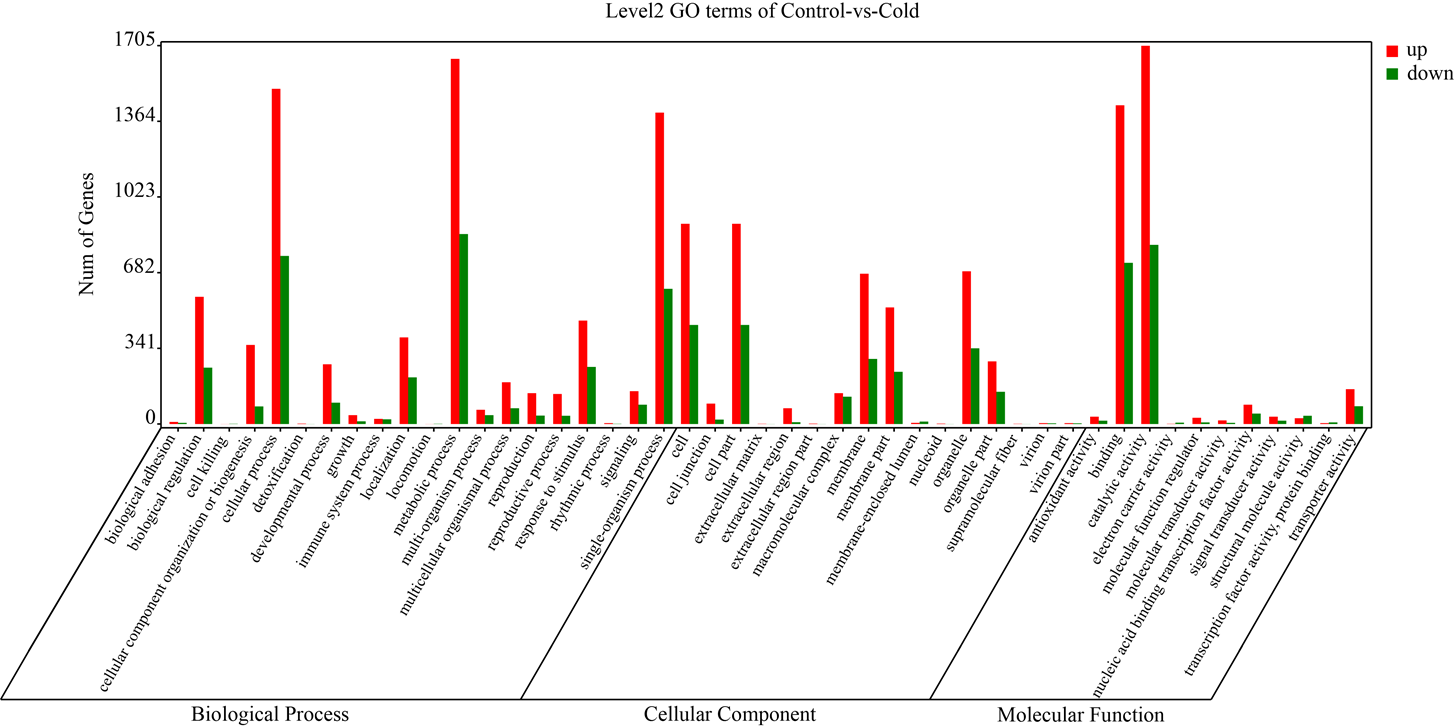


**Figure S2. Gene ontology (GO) term enrichment based on DGE analysis.**

**
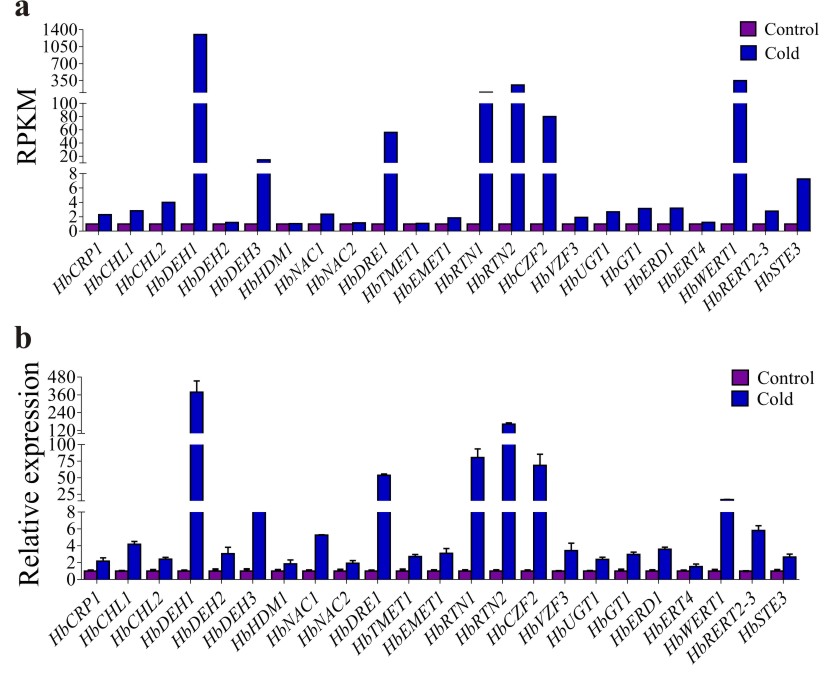
**

**Figure S3. qRT-PCR validation of differential gene expression after cold treatment.** Expression patterns of one cold-regulated plasma membrane protein gene (HbCRP1), two chloroplast genes (HbCHL1/2), three dehydratase genes (HbDEH1/2/3), one histone demethylase gene (HbHDM1), two zinc finger protein genes (HbCZF2/VZF3), two methyltransferase genes (HbTMET1/EMET1), one sugar transporter gene (HbSTE3), two glycosyltransferase genes (HbUGT1/GT1), one early-responsive to dehydration stress gene (HbERD1), two nac domain-containing protein genes (HbNAC1/NAC2), one dehydration-responsive element-binding protein gene (HbDRE1), two transposon genes (HbRTN1/2), and three ethylene-responsive transcription factor genes (HbERT4/WERT1/RERT2-3) were determined by digital gene expression analysis (a), and by qRT-PCR (b). RPKM, reads per kilobase million. Error bars represent the standard errors of the means of three independent replicates.

**Table S1.** **Statistics of filtering clean reads in RRBS.**

|  | **Before filtering** | | **After filtering** | |  |  |  |  |
| --- | --- | --- | --- | --- | --- | --- | --- | --- |
| **Samples** | **clean Reads** | **clean data（bp）** | **clean Reads** | **clean data（bp）** | **Q20** | **Mapped Reads** | **Mapped Ratio(%)** | **Sequence Depth** |
| Control | 83531456 | 8100345025 | 82143730 | 7900525750 | 0.9785 | 74950281 | 0.9124 | 6.82 |
| Cold | 82345180 | 7966520637 | 81565518 | 7766520664 | 0.9784 | 77324688 | 0.948 | 7.04 |

**Table S2. Statistics of RNA-Sequencing data.**

| **Samples** | **Before Filter** | |  |  |  | **After Filter** | |  |  |  |  | |
| --- | --- | --- | --- | --- | --- | --- | --- | --- | --- | --- | --- | --- |
|  | **Clean Data(bp)** | **Q20(%)** | **Q30(%)** | **N(%)** | **GC(%)** | **HQ Clean Data(bp)** | **Q20(%)** | **Q30(%)** | **N(%)** | **GC(%)** |  | |
| Control | 3811881600 | 3635256772 (95.4%) | 3425926388 (89.9%) | 68017 (0.0%) | 1610977318 (42.3%) | 3764205386 | 3605959844 (95.80%) | 3404257835 (90.4%) | 18947 (0.00%) | 1589753031 (42.2%) |  |  |
| Cold | 3474151800 | 3314668319 (95.4%) | 3122326821 (89.9%) | 62432 (0.0%) | 1481922593 (42.3%) | 3433710574 | 3289849930 (95.81%) | 3104029977 (90.4%) | 16535 (0.00%) | 1463679214 (42.6%) |  |  |
| Clean Data (PE) = Clean Reads1 Num x Read1 length + Clean Reads2 Num x Read2 length;  Clean Data (SE) = Clean Reads1 Num x Read1 length | | | | | | | | | | |  |  |
| HQ Clean Data (PE) = HQ Clean Reads1 Num x Read1 length + HQ Clean Reads2 Num x Read2 length;  HQ Clean Data (SE) = HQ Clean Reads1 Num x Read1 length. | | | | | | | | | | |  |  |

**Table S3. Statistics of sequencing** **coverage** **in genomic functional elements by RRBS.**

| **Samples** | **region** | **C** | | | **CG** | | | **CHG** | | | **CHH** | | |
| --- | --- | --- | --- | --- | --- | --- | --- | --- | --- | --- | --- | --- | --- |
|  |  | **Total** | **Coverage** | **Coverage** | **Total** | **Coverage** | **Coverage** | **Total** | **Coverage** | **Coverage** | **Total** | **Coverage** | **Coverage** |
|  |  | **sites** | **sites** | **rate(%)** | **sites** | **sites** | **rate(%)** | **sites** | **sites** | **rate(%)** | **sites** | **sites** | **rate(%)** |
| Control | Genebody | 3,798,581 | 1,352,838 | 35.61 | 269,511 | 109,674 | 40.69 | 586,469 | 219,699 | 37.46 | 2,942,599 | 1,023,465 | 34.78 |
|  | Exon | 1,522,543 | 633,367 | 41.6 | 134,497 | 63,687 | 47.35 | 269,603 | 114,723 | 42.55 | 1,118,441 | 454,957 | 40.68 |
|  | Intron | 2,113,288 | 673,989 | 31.89 | 123,213 | 42,140 | 34.2 | 295,448 | 98,604 | 33.37 | 1,694,627 | 533,245 | 31.47 |
|  | CDS | 1,216,483 | 506,642 | 41.65 | 108,157 | 51,232 | 47.37 | 221,532 | 94,370 | 42.6 | 886,794 | 361,040 | 40.71 |
|  | 5’_UTR | 142,060 | 63,031 | 44.37 | 15,772 | 8,051 | 51.05 | 22,280 | 10,213 | 45.84 | 104,006 | 44,767 | 43.04 |
|  | 3’_UTR | 183,714 | 73,158 | 39.82 | 12,406 | 5,422 | 43.7 | 29,378 | 11,889 | 40.47 | 141,930 | 55,847 | 39.35 |
| Cold | Genebody | 3,798,581 | 921,435 | 24.26 | 269,511 | 81,166 | 30.12 | 586,469 | 152,471 | 26 | 2,942,599 | 687,798 | 23.37 |
|  | Exon | 1,522,543 | 445,615 | 29.27 | 134,497 | 48,234 | 35.86 | 269,603 | 81,276 | 30.15 | 1,118,441 | 316,105 | 28.26 |
|  | Intron | 2,113,288 | 445,066 | 21.06 | 123,213 | 30,201 | 24.51 | 295,448 | 66,718 | 22.58 | 1,694,627 | 348,147 | 20.54 |
|  | CDS | 1,216,483 | 358,549 | 29.47 | 108,157 | 39,047 | 36.1 | 221,532 | 66,996 | 30.24 | 886,794 | 252,506 | 28.47 |
|  | 5’_UTR | 142,060 | 44,940 | 31.63 | 15,772 | 6,043 | 38.31 | 22,280 | 7,375 | 33.1 | 104,006 | 31,522 | 30.31 |
|  | 3’_UTR | 183,714 | 48,695 | 26.51 | 12,406 | 3,860 | 31.11 | 29,378 | 8,130 | 27.67 | 141,930 | 36,705 | 25.86 |

**Table S4. Statistic of average levels of methylation revealed by RRBS.**

| **Samples** | **C(%)** | **CG(%)** | **CHG(%)** | **CHH(%)** |
| --- | --- | --- | --- | --- |
| Control | 20.48 | 64.68 | 55.37 | 7.29 |
| Cold | 19.12 | 59.8 | 49.88 | 5.92 |

**Table S5.** **Statistics of mapping genes of RNA-Seq reads against Reyan7-33-97.**

| **Samples** | **Known Gene Num** | **New Gene Num** | **All Gene Num** |
| --- | --- | --- | --- |
| Control | 28052 (63.93%) | 1079 | 29131 |
| Cold | 29154 (66.44%) | 1088 | 30242 |

Known Gene: the genes have been previously predicted in genomic sequence.

New gene: the genes have not been predicted in genomic sequence so far.

**Table S6. Primers used for quantitative real-time PCR**

| Primer name | Primer sequence (5’ to 3’) |
| --- | --- |
| Hb18SqRT-F  Hb18SqRT-R  HbRH8qRT-F  HbRH8qRT-R  Hb7aqRT-F  Hb7aqRT-R  Hb7bqRT-F  Hb7bqRT-R  QHbCRP2-F  QHbCRP2-R  QHbDEH1-F  QHbDEH1-R  QHbHDM1-F  QHbHDM1-R  QHbDRE1-F  QHbDRE1-R  QHbCHL1-F  QHbCHL1-R  QHbRTN1-F  QHbRTN1-R  QHbERT4-F  QHbERT4-R  QHbDEH2-F  QHbDEH2-R  QHbVZF3-F  QHbVZF3-R  QHbTMET1-F  QHbTMET1-R  QHbNAC2-F  QHbNAC2-R  QHbUGT1-F  QHbUGT1-R  QHbSTE3-F  QHbSTE3-R  QHbRTN2-F  QHbRTN2-R  QHbWERT1-F  QHbWERT1-R  QHbDEH3-F  QHbDEH3-R  QHbCZF2-F  QHbCZF2-R  QHbEMET1-F  QHbEMET1-R  QHbNAC1-F  QHbNAC1-R  QHbGT1-F  QHbGT1-R  QHbERD1-F  QHbERD1-R  QHbCHL2-F  QHbCHL2-R  QHbRERT2-3-F  QHbRERT2-3-R | GCTCGAAGACGATCAGATACC  TTCAGCCTTGCGACCATAC  TCACAGGGTTGGTAGATCAG  CCAAGCTCTTGCTCAATCC  CACCACCAGAGAGAAAGTACAG  GATGGACCAGACTCATCGTATTC  CAGTGTCTGGATAGGAGGATCTA  AAATGGACCGGACTCATCATAC  CTTATTCCTCAGCCTGT  GAGCTAGATTGCGTTTG  CTCCCTGCTATTGTTTC  CCTCATAGTCCATTTCG  GATTCTTGCTCGCTATG  CTAGCAACCAACCTTAC  GAGGTTGAATTGGAACG  CCACATCAATACAGGGA  CACTGATTGGCGAAGAA  GACACCCACCCAATATAC  GGGCAAGGTGTAATATG  TTCCCTCAGATCTTTCC  TGGATCTTAACCTACCC  GTCTCGACTGGAAATTG  CCTGTACGGATTACCTA  GATTCGACCTTGTCTTG  GTGACAGAGCTTATGTG  AACTTCTCTACCAGGAG  CAGAGCCATACATGAAG  CTCACCAGAACTCTTTG  CATGTACCTACAGAAGC  CTATACCCAAACCCATC  CTCCAGGAAATGCTAAG  GAAGTTGAGAGGTGTTC  TACAGTCCCTTCTCTAC  CTATGCACTCTCTTTCC  CACCTCCTGTGTTCAAT  GGTAGTCTTCCTCAACAG  CACCCAAAGGGAACAAA  GAGCATGCAGAAGGTTT  CATTGAGGAGATTGGAG  CCTATCATTGGGACTTG  AGGAAGTACCACTACTC  GAAGCCAGATCTCAAAC  GGTGACTACGTCAAATG  CAAGATCCTCCTCCTAA  GTGACTGCATAGGATTC  GGTGAACCTGCTAAATC  CTGAAACCTCATCTGTC  CCATCATCGTTCTTCTC  GTGATGATGACGAAGAG  CTGCTAATTCTGCTAGG  GGACCTGGTTTATTTCC  GAGAAGTGTCGCAATAG  AACCCTATAGACGTCAC  TAGCTAAGGCGAGTAAG |
